# Oxygen in Therapeutics

**Published:** 1887-09

**Authors:** 


OXYGEN IN THERAPEUTICS.—A treatise explaining the apparatus, the material, and the processes used in the preparation of oxygen and other gases, with which it may be combined; also its administration and effects, illustrated by clinical experience of the author and other. By C. E. Ehninger, M. D. Illustrated sexto., pp. 157. Chicago, W. A. Chatterton & Co., 1887.

Now that the subject of oxygen in Therapeutics is attracting so much attention, this book is timely, and will be read with interest and profit, though, we must admit, it does not quite come up to our expectations. It may be that the subject is too new,—i. e.—as a rational remedy—in the hands of the regular profession—for, heretofore, and for many years, it has been considered the property of the irregulars; and that a second edition will be an improvement, when something more definite shall have been learned, and more, and better records of observations shall have been made.

---

WANTED: A few copies of our last edition, August, 1887. The demand was tremendous, and we robbed our file to accommodate new subscribers; subscriptions have literally poured in since July 1. Friends having duplicates will oblige by letting us know,—will buy a small number.

